# Normative anthropometry and proportions of the Kenyan-African face and comparative anthropometry in relation to African Americans and North American Whites

**DOI:** 10.1186/s40902-019-0191-7

**Published:** 2019-02-22

**Authors:** Saurab S. Virdi, David Wertheim, Farhad B. Naini

**Affiliations:** 1 Department of Orthodontics, St George’s Hospital and King’s College London, London, UK; 2 Faculty of Science, Engineering and Computing, Kingston University, London, UK; 3 Kingston and St George’s Hospitals and St George’s Medical School, London, UK

**Keywords:** Craniofacial anthropometry, Kenyan, Proportions, Normative values

## Abstract

**Background:**

There is no normative craniofacial anthropometric data for the Kenyan-African population. The purpose of this investigation was to determine normative anthropometric craniofacial measurements and proportional relationships for Kenyans of African descent and to compare the data with African Americans (AA), North American Whites (NAW), and neoclassical canons.

**Methods:**

Twenty-five direct facial anthropometric measurements, and 4 angular measurements, were taken on 72 Kenyan-African participants (age range 18–30 years) recruited at the University of Nairobi in Kenya. The data were compared with AA and NAW populations, and neoclassical canons. Descriptive statistics of the variables were computed for the study population.

**Results:**

Significant differences between both Kenyan males and females were detected in forehead height (~ 5 mm greater for males, ~ 4.5 mm for females), nasal height (reduced by ~ 4 mm in males, ~ 3 mm in females), nasal width (8–9 mm greater), upper lip height (> 3 mm), and eye width (greater by ~ 3 mm) compared to NAW subjects. All vertical measurements obtained were significantly different compared with NAW. Differences were observed in comparison with AA subjects, but less marked. Mouth width was similar in all groups. Angular measurements were variable. Neoclassical canons did not apply to the Kenyan population.

**Conclusions:**

Anthropometric measurements of NAW showed clear differences when compared with the Kenyan population, and variations exist with comparative AA data. The anthropometric data in terms of linear measurements, angular measurements, and proportional values described may serve as a database for facial analysis in the Kenyan-African population.

## Background

Many patterns of growth, development, and treatment changes may be recorded with good levels of precision using anthropometry [1–5]. The first study to test the pertinence of neoclassical facial canons included samples of 6-, 12-, and 18-year-old North American Caucasians [4]. Over the years, the appropriateness of these canons has been tested in other ethnic groups including Indian [6, 7], African-American [8], Turkish [9], Vietnamese, Thai, and Chinese populations [10]. These anthropometric studies were performed by means of using direct manual methodology, such as spreading and sliding calipers, and have permitted the evaluation of numerous craniofacial measurements in various ethnic groups [11]. However, data on Kenyans of African descent is inadequate [12].

Anthropometric information provides useful data on the distribution of numerous measurements of human subjects, enabling the impartial appraisal of outcomes before and after treatment [13, 14]. Craniofacial anthropometry is an uncomplicated, economical, effective, and non-invasive process for quantitative analysis of craniofacial morphology and it involves taking direct clinical measurements such as linear distances, proportions, angles, and ratios [15]. Craniofacial anthropometry is appropriate for population studies because of the accessibility of comparative and conventional databases [15].

**Fig. 1:**
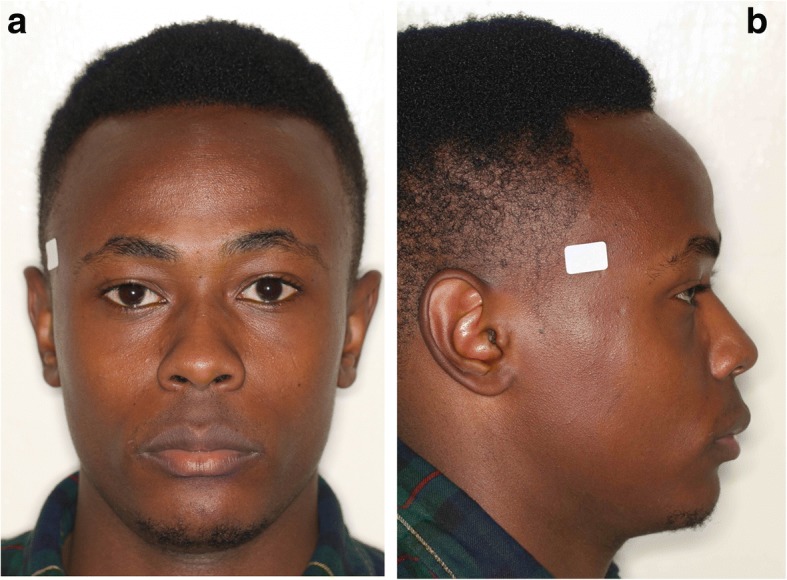
**a** Frontal and **b** profile views of Kenyan African male

An extensively utilized collection of anthropometric measurements, comprising of 47 surface landmarks (Fig. 1), to develop facial canons in order to help in analyzing and describing the faces of North American Caucasians have been described [16]. These canons were subsequently tested on a variety of ethnic groups with participants from 13 European countries (Azerbaijan, Bulgaria, Croatia, Czech Republic, Germany, Greece, Hungary, Italy, Poland, Portugal, Russia, Slovakia, and Slovenia), 3 Middle Eastern countries (Egypt, Iran, and Turkey), 5 East Asian countries (India, Japan, the Chinese of Singapore, Vietnam, and Thailand), 3 African states (Angola, Tonga, and Zulu), and African Americans from the USA [16].

An investigation compared the Sudanese female (SF) face with those of African Americans (AA) and North American whites (NAW) and recognized differences, expressing that the neoclassical norms were unreliable guides to the SF face as they were considerably taller and narrower than the AA or NAW female face respectively [11]. Another investigation recognized that the typical AA female does not fit the neoclassical criterion of facial proportions, and varied considerably in the horizontal dimension measurements when compared to those of white subjects [17].

Photogrammetric analysis may be less accurate than anthropometric analysis [18]. Nevertheless, an investigation comprising the angular photogrammetric comparison of soft tissue profiles of 177 black Kenyans and 156 Chinese was undertaken, which established numerous contrasts in the typical angular measurements of facial profiles between black Kenyans, Chinese, and white standards [12]. Jeffries et al. [19] photogrammetrically examined 200 AA participants and compared the results with those of Farkas et al. [14]. They determined that AA and white participants had comparable vertical facial proportions, though the horizontal proportions varied considerably and were in accordance with previously published data [19]. Two investigations have noted that the South Indian population, in general, had a wider lower face while NAW showed wider midface and overall greater values of proportional indices than North American Caucasian population [6, 7]. A Turkish population study clearly shows anthropometric variation for fronto-occipital, circumference, intercanthal distance, outer canthal distance, near and distant interpupillary distance, canthal index, and circumference-interorbital index with age [9].

Normative craniofacial anthropometric values (linear, angular, and proportional) aid in diagnostic determination and treatment planning for patients, who come from diverse ethnic backgrounds and have need for esthetic and reconstructive dentofacial or craniofacial surgery. A database of normative values for each ethnic group is essential. Universally applied criteria of esthetic attractiveness and proportions may be misleading, due to ethnic variation, and dependence on neoclassical proportional canons, may be equally spurious [1]. To date, normative anthropometric data and comparative information that could be used for treatment planning in craniofacial and orthognathic surgery has been inaccessible for Kenyans of African descent. The proposed investigation aimed to gather the required normative data, and to assess the differences in facial proportions between Kenyan participants compared to those of African Americans (AA), North American Whites (NAW), and neoclassical canons.

## Methods

### Subjects and materials

Ethical approval was obtained from the University of Nairobi Ethics and Research Committee. The sample size was determined using simple sampling method based on previous anthropometric investigations [11, 17]. This prospective cross-sectional investigation was undertaken at the University of Nairobi in Kenya.

The inclusion criteria were:

• Male and female participants (> 18 years of age) studying at the University of Nairobi.

• Being of Kenyan descent (each participant was questioned regarding their family background and both sets of grandparents determined to be of Kenyan descent).

• No history of previous facial surgical procedure.

• Having average/normal facial appearance (as visually assessed by the lead investigator).

Each invited participant was provided with an information sheet and verbal information, and informed consent was obtained.

Ethical approval was granted by the Ethics and Research Committee, University of Nairobi (ref: KNH-ERC/A/289).

### Measurements and technique

Subsequently, anthropometric measurements were taken with a digital vernier caliper, followed by frontal and profile facial photographs taken in a natural head position. A sliding digital vernier caliper was used to measure predetermined anthropometric facial parameters directly on each subject. These measurements were performed in agreement with well-established methods previously described [13]. The frontal and profile photograph of each participant was taken utilizing a standardized method with the participant in natural head position, the same camera to participant distance, the same background, and comparable illumination by means of a digital camera, a Canon 70D (with macro lens 100 and Macro Ring Flash II).

All measurements were collected by one author (SSV) with the subjects’ head in natural head position, and recorded in millimeters.

**Fig. 2:**
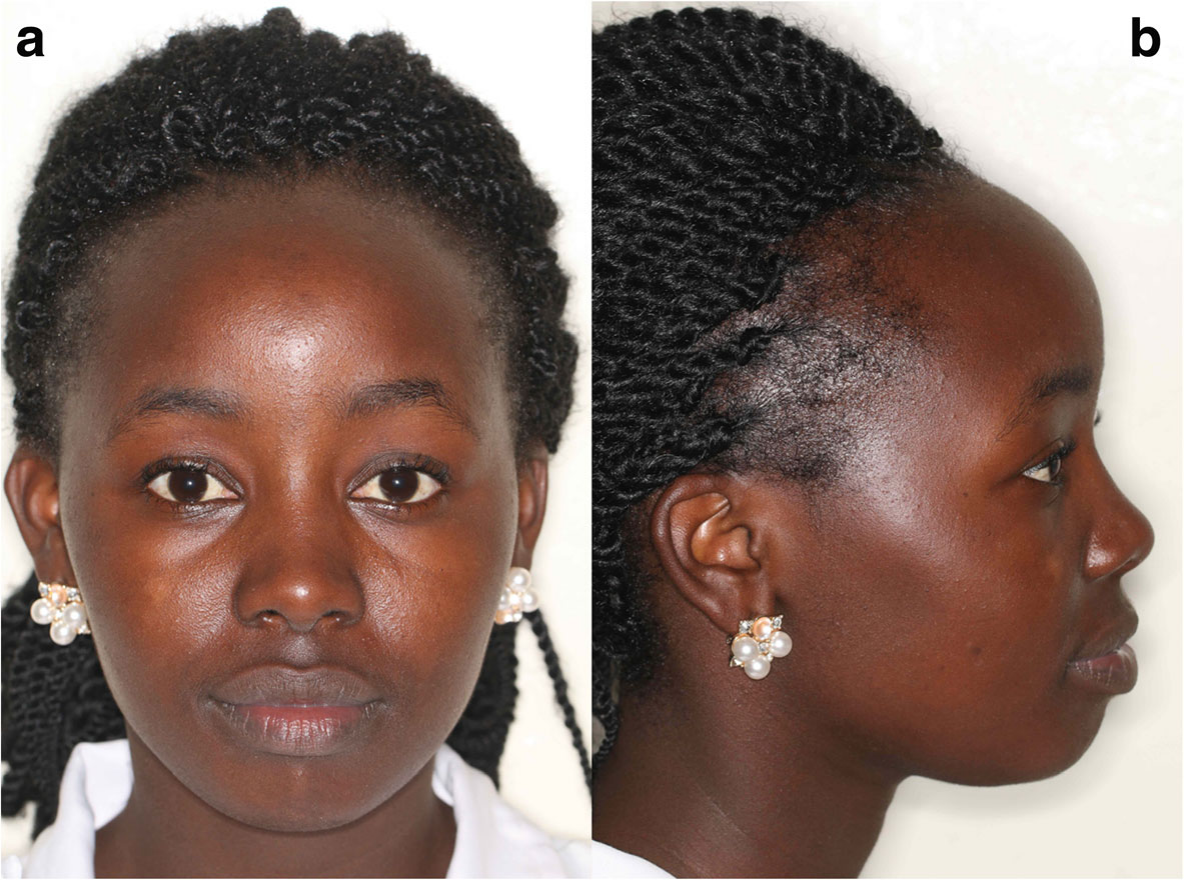
**a** Frontal and **b** profile views of Kenyan African female

**Fig. 3:**
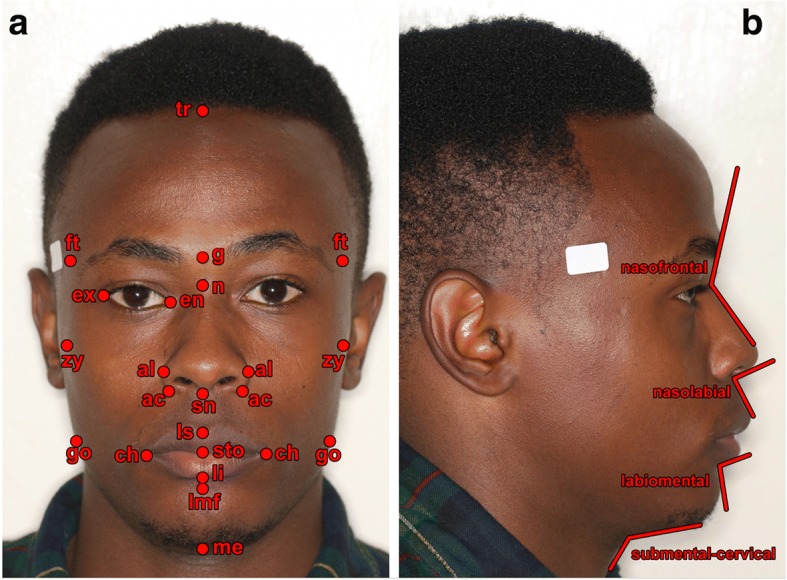
**a** Frontal view demonstrating principal soft tissue landmarks used for linear measurements: *tr* trichion, *g* glabella, *n* nasion, *sn* subnasale, *ls* labrale superius, *sto* stomion, *li* labrale inferius, *lmf* labiomental fold, *me* menton, *ft*, frontotemporale, *zy* zygion, *go* gonion, *ex* exocanthion, *en* endocanthion, *al* alare, *ac* alar curvature point, *ch* cheilion. **b** Profile view demonstrating angular measurements

Figures 1 and 2 demonstrate examples of a male and a female Kenyan subject participating in this investigation. Figure 3 illustrates the principal facial soft tissue landmarks, permitting the linear and angular measurements used in this investigation.

The following were the principal measurements undertaken (Fig. 3):

- Head: tr-n and tr-g (forehead height)
- Orbits: en-en (intercanthal distance), ex-ex (biocular width), en-ex (eye fissure length)
- Face: ft-ft (bitemporal width), zy-zy (bizygomatic face width), go-go (bigonial width), tr-me (physiognomical face height), n-me (morphological face height), g-sn (midface height), sn-me (lower face height
- Nose: al-al (Morphological nose width), ac-ac (nasal alar base width), n-sn (nose height)
- Labio-oral region: ch-ch (mouth width), sn-ls (philtrum height), sn-sto (upper lip height), ls-sto (upper vermilion height), sto-li (lower vermilion height), sto-lmf (lower lip height), lmf-me (chin height)
- Angular measurements: nasofrontal, nasolabial, labiomental, and submental-cervical.

### Measurement error and reliability

An intra-examiner reliability test was performed with five subjects and their measurements recorded at two different times, 2 weeks apart.

### Statistical analysis

**Fig. 4:**
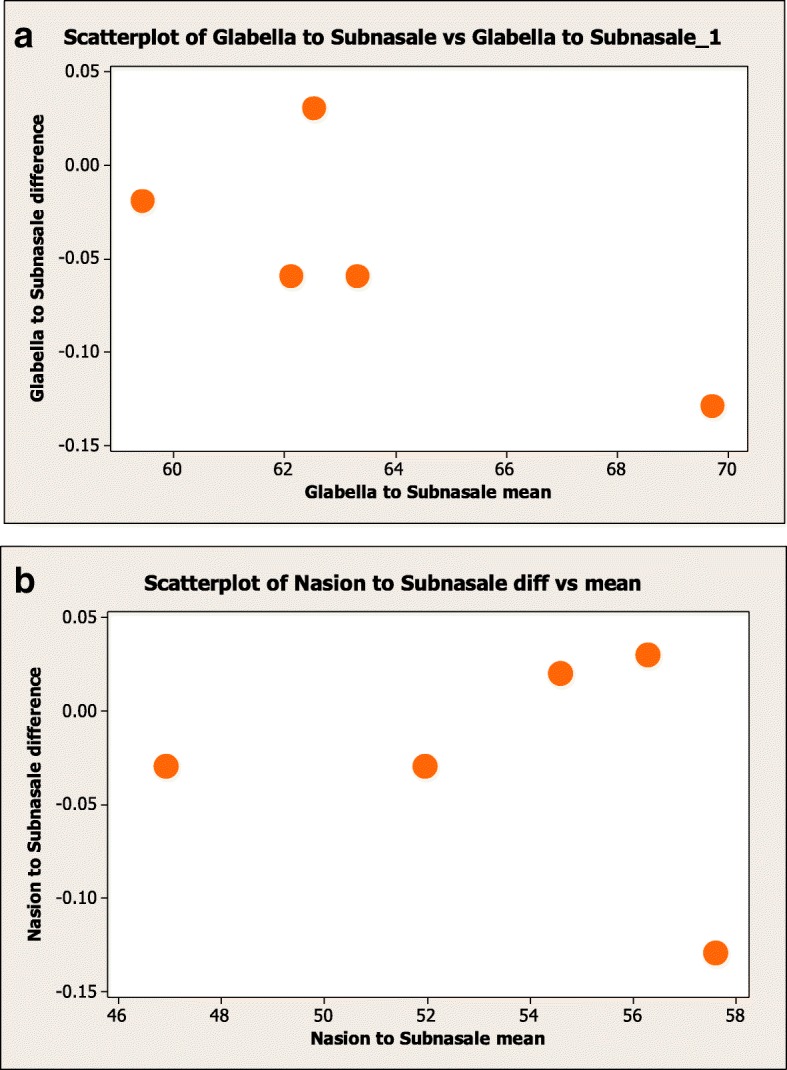
Scatterplot of vertical facial measurements, permitting examination of trends in the relationships, and changes in spread of one variable as a function of the other. **a** Glabella to subnasale. **b** Nasion to subnasale

Data analysis was undertaken using Microsoft Excel 2010 (Microsoft Corporation, USA) and Minitab version 16 (Minitab Inc., USA) software for Windows. Descriptive statistics of the variables were computed for the study population. Two-sample *t* tests were used to compare the distribution means of ten measurements, horizontal and vertical with published NAW and AA data [8, 20]. For some measurements, there was insufficient data available to compare using the two-sample *t* tests; hence one-sample *t* tests were used to compare these data from the Kenyan participants with the North American white and African American mean values, to provide an indication of differences. Intraexaminer reliability was analyzed using the formula proposed by Dahlberg that determined method error (ME) = √∑(*x*_1_ – *x*_2_)^2^/2*n* where *x*_1_ is the first measurement, *x*_2_ the second measurement, and *n* is the number of repeated records. Measurements of five participants’ were repeated at an interval of 2 weeks to enable assessment of repeatability (Fig. 4).

## Results

### Examiner reliability

The results of intraexaminer reliability were calculated using the Bland-Altman test, Dahlberg method, and absolute difference divided by the mean. All the methods showed a low method error that was generally less than 0.5 mm for linear measurements (vertical and horizontal) and < 2.4° for angular measurements, which is considered acceptable (Fig. 1, Table 1).

**Table 1:** Dahlberg coefficient of horizontal and vertical measurements

Vertical measurements	Dahlberg coefficient	
Trichion to Glabella	0.041	
Trichion to Nasion	0.083	
Glabella to Subnasale	0.050	
Nasion to Subnasale	0.045	
Subnasale to Soft tissue menton	0.018	
Upper lip height	0.046	
Lower lip height	0.086	
Philtrum height	0.050	
Lateral commissure height	0.043	
Vermillion height Upper	0.039	
Vermillion height Lower	0.033	
Chin height	0.058	
Lower lip to labiomental fold (LMF)	0.339	
LMF (depth)	0.043	
Lower lip to Soft tissue menton	0.076	
Horizontal measurements	Dahlberg coefficient	Mean (abs diff/mean) (%)
Ex-Ex	0.036	0.053
Medial canthus to lateral canthus (ex-en)	0.024	0.101
En-En	0.022	0.050
Right ala to left ala (al-al)	0.029	0.063
Right ala curvature to left ala curvature (ac-ac)	0.235	0.993
Mouth width (ch-ch)	0.054	0.105
Bitemporal width (ft-ft)	0.027	0.042
Bizygomatic width (zy-zy)	0.575	0.395
Bigonial width (go-go)	0.024	0.027

### Principal measurements

Descriptive statistics for vertical and horizontal measurements for the Kenyan male and female are given in Tables 2 and 3. Tables 4 and 5 show the angular measurements of the Kenyan male and female face compared with the North American White (NAW) and African American (AA) populations.

**Table 2:** Comparison with Farkas et al. [8] data using two-sample *t* tests (Kenyan males)

Kenyan male face					
	KM Mean (*n* = 36)	NAW (SD) (*n*-109)	*P* value	AA (SD) (*n* = 50)	*P* value
Vertical measurements					
Forehead height II tr-n	72.2 (2.3)	67.1 (7.5)	< 0.001*	72.0 (7.7)	0.850
Nasal height n-sn	51.0 (1.9)	54.8 (3.3)	< 0.001*	51.9 (3.0)	0.077
Lower face height sn-me	76.4 (3.6)	72.6 (4.5**)**	< 0.001*	78.9 (6.7)	0.025
Upper lip height sn-sto	25.5 (1.3)	22.3 (2.1)	< 0.001*	26.1 (2.5)	0.107
Lower lip height sto-sl	22.5 (1.9)	19.7 (2.1)	< 0.001*	22.5 (1.9)	0.402
Horizontal measurements					
Intercanthal distance en-en	32.2 (1.9)	33.3 (2.7)	0.011	35.8 (2.8)	< 0.001*
Eye width ex-en	34.0 (3.4)	31.3 (1.3)	< 0.001*	32.9 (1.7)	0.094
Biocular width ex-ex	98.2 (3.5)	91.2 (3.0)	< 0.001*	96.8 (4.5)	0.110
Nasal width al-al	43.2 (3.8)	34.9 (2.1)	< 0.001*	44.1 (3.4)	0.234
Mouth width ch-ch	55.9 (3.3)	54.5 (3.0)	0.027	54.6 (4.1)	0.106

*Clinically significant difference set at +/− 3 mm

**Table 3:** Comparison with Farkas et al. [8] data using two-sample *t* tests (Kenyan females)

Kenyan female face					
	KF mean (*n* = 36)	NAW (SD) (*n*-200)	*P* value	AA (SD) (*n* = 50)	*P* value
Vertical measurements					
Forehead height II tr-n	67.5 (2.9)	63.0 (6.0)	< 0.001*	67.1 (5.9)	0.693
Nasal height n-sn	47.6 (3.1)	50.6 (3.1)	< 0.001*	48.8 (3.7)	0.114
Lower face height sn-me	69.5 (4.8)	64.3 (4.0**)**	< 0.001*	71.5 (5.2)	0.061
Upper lip height sn-sto	24.0 (2.5)	20.1 (2.0)	< 0.001*	24.5 (3.0)	0.435
Lower lip height sto-sl	20.7 (1.1)	17.8 (4.7)	< 0.001*	20.2 (2.4)	0.163
Horizontal measurements					
Intercanthal distance en-en	32.1 (1.4)	31.8 (2.3)	0.225	34.4 (0.5)	< 0.001*
Eye width ex-en	33.7 (1.5)	30.7 (1.2)	< 0.001*	32.2 (2.0)	0.087
Biocular width ex-ex	94.4 (4.9)	87.8 (3.2)	< 0.001*	92.9 (5.3)	0.185
Nasal width al-al	40.7 (3.7)	31.4 (2.0)	< 0.001*	40.1 (3.2)	0.411
Mouth width ch-ch	52.0 (4.0)	50.2 (3.5)	0.012	53.6 (4.0)	0.073

*Clinically significant difference set at ± 3 mm

**Table 4:** Angular measurements: two-sample *t* test for Kenyan males

Kenyan male (*n* = 34)	Mean	SD	NAW mean (*n* = 50)	SD	*P* value	AA mean (*n* = 50)	SD	*P* value
Nasofrontal	127.3	9.0	130.3	7.4	0.107	126.5	12.0	0.741
Nasolabial	85.5	10.1	99.8	11.8	0.001	71.4	14.5	0.001
Labiomental	128.2	10.8	113.5	20.7	0.001	101.5	17.7	0.001
Submental-cervical	109.1	14.5

**Table 5:** Angular measurements: two-sample *t* test for Kenyan females

Kenyan female(*n* = 36)	Mean	SD	NAW mean (*n* = 50)	SD	*P* value	AA mean (*n* = 50)	SD	*P* value
Nasofrontal	127.9	3.0	134.3	7.0	0.001	127.6	8.1	0.786
Nasolabial	85.2	13.8	104.2	9.8	0.001	73.9	14.5	0.001
Labiomental	116.9	21.1	121.4	14.4	0.269	101.6	18.0	0.001
Submental-cervical	109.1	14.5						

In view of multiple comparisons, the level taken for significance in these studies was *p* < 0.001. For five vertical and five horizontal measurements, and three angular measurements, all the comparative data were available from Farkas et al. [8] thus allowing analysis using two-sample *t* tests. Clinically significant differences were based on the ability to detect a difference greater than 3 mm between the two equal halves of the face. Farkas et al. [8] considered differences of up to 2 mm to be within normal range, hence the cut-off minimum was set at 3 mm.

To date, only one study has suggested a threshold of a clinically relevant difference expressed in degrees [21]. In this investigation, the clinically significant difference was set at 10°, in order to demonstrate differences that were clinically significant and visually identifiable, perhaps even to the untrained eye.

### Specific Kenyan-African craniofacial data

The following results were obtained:

Head: Forehead height (tr-n) of all the Kenyan males and females were increased compared to the North American whites (*p* < 0.001) (clinically significant > 3 mm) and were similar to the African American population (male *p* = 0.85 and female *p* = 0.693) (Tables 2 and 3).

Labio-oral region: The mouth width (ch-ch) was larger by 1.4 mm compared with NAW, and 1.3 mm to AA, though not clinically significant, (*p* = 0.027) and (*p* = 0.106), respectively. Similarly, the Kenyan female had a greater mouth width (1.8 mm, *p* = 0.012) compared with NAW, though was smaller compared with AA (− 1.6 mm); this was not statistically significant (*p* = 0.073) (Tables 2 and 3).

Facial region: Of the ten measurements tested, the five vertical ones (tr-n, n-gn, sn-me, sn-sto, sto-sl) showed a significant difference (*p* < 0.001) compared with those of NAW and were similar to AA in both sexes, with no statistical difference detected (Tables 2 and 3).

Orbits: Eye fissure (en-ex) was increased in the Kenyan male compared with the NAW by 2.65 mm (*p* < 0.001), and the AA by 1.1 mm (*p* = 0.094). A similar result was observed in the Kenyan females compared with NAW and AA with a mean difference of 3.0 mm (*p* < 0.001) and 1.5 mm (*p* < 0.001), respectively (Tables 2 and 3). The biocular width (ex-ex) was significantly greater compared with the NAW with a mean difference of 7 mm (*p* < 0.001) in the males and 6.6 mm (*p* < 0.001) in the females. The differences were not significant when compared with the AA group (*p* = 0.110 in males and *p* = 0.185 in females) though the Kenyan females had a greater mean difference of 1.4 mm compared with the AA population (Tables 2 and 3).

**Nose**: Nose height (n-sn) was reduced by a mean difference of 3.8 mm (*p* < 0.001) in the Kenyan male and 3.0 mm (*p* < 0.001) in Kenyan females compared with the NAW. Both males and females had slightly reduced mean height differences compared to the AA, though this was not significant (*p* = 0.077 in males, *p* = 0.114 in females). However, nasal width (al-al) was greater and extremely significant with a mean difference of 8.3 mm (*p* < 0.001) in males and 9.3 mm (*p* < 0.001) in females. These measurements were almost identical when compared with the AA populations, with a mean difference of − 1.0 mm (*p* = 0.234) in the males and 0.6 mm (*p* = 0.411) in the females (Tables 2 and 3). The nasolabial and labiomental angular measurements of the Kenyan African male had a clinically significant difference of 10° when compared with the North American white and the African American subjects (Table 4). In the Kenyan females, only the nasolabial angle had a clinically significant difference when compared with the NAW and AA populations. No significant difference was observed when compared to NAW, though a clinical significance was observed in the labiomental angle when compared to the AA female (Table 5).

### Comparative craniofacial data

#### Comparative data with NAW and AA

For all the above measurements, there were clear clinically significant differences between Kenyan cohort male subjects compared with NAW (*p* < 0.001), except for en-en (*p* = 0.011) and mouth width (ch-ch) (*p* = 0.027). In contrast, when comparing with the AA male cohort, there were no clinically significant differences, except the intercanthal distance (en-en) (*p* < 0.001). Similar results were obtained with the Kenyan female data, with all measurements demonstrating clinically significantly differences from NAW, except en-en (*p* = 0.225), mouth width (ch-ch) (*p* = 0.012, borderline), and compared with AA no clinically significant differences except a marginal intercanthal distance difference (en-en) (*p* < 0.001).

In terms of differences compared to the mean (one-sample *t* test), when comparing the Kenyan male face to NAW, of the 22 measurements taken, 10 of the vertical measurements and 5 of the horizontal measurements showed statistically significant differences. Clinical significant differences were observed in nine vertical measurements and four horizontal measurements (Table 6).

**Table 6:** Comparison of the average facial measurements of the Kenyan male (KM) face and Kenyan female (KF) face with North American whites. NAW data from Farkas et al. [8, 14, 20] and the African-American data from Farkas et al. [8] using one-sample *t* test

	KM mean (SD) (*n* = 36)	NAW mean (SD) difference (*n* = 109)	*P* value	AA mean (SD) difference (*n* = 50)	*P* value
Kenyan male face					
Vertical measurements					
Forehead height I (tr-g)	61.4 (3.2)	4.4 (3.2)	< 0.001	− 0.4 (3.2)	0.496
Forehead height II (tr-n)	72.2 (2.3)	5.1 (2.3)	< 0.001	0.2 (2.3)	0.576
Midface height (g-sn)	64.8 (6.5)	− 2.4 (6.5)	0.033	− 4.0 (6.5)	0.001
Nasal height (n-sn)	51.0 (1.9)	− 3.8 (1.9)	< 0.001	− 0.9 (1.9)	0.005
Lower face height (sn-me)	76.4 (3.6)	3.8 (3.6)	< 0.001	− 2.6 (3.6)	< 0.001
Upper lip height (sn-sto)	25.5 (1.3)	3.1 (1.3)	< 0.001	− 0.7 (1.3)	0.004
Lower lip height (sto-sl)	22.5 (1.9)	2.8 (1.9)	< 0.001	0.4 (1.9)	0.230
Philtrum height (sn-ls)	15.5 (1.3)	− 0.4 (1.3)	0.072	− 0.9 (1.3)	< 0.001
Lateral commissure height	23.5 (2.6)				
Upper vermillion height (Is-sto)	13.7 (1.3)	5.9 (1.3)	< 0.001	0.1 (1.3)	0.726
Lower vermillion height (sto-li)	13.8 (0.9)	4.5 (0.9)	< 0.001	0.0 (0.9)	0.903
Chin height (fml-me)	36.5 (3.1)	3.4 (3.1)	< 0.001	0.5 (3.1)	0.307
Lower lip to labiomental fold	12.1 (1.9)	0.2 (1.9)	0.473	0.3 (1.9)	0.021
Labiomental fold	8.2 (1.9)				
Lower lip to menton	56.2 (3.5)	8.2 (3.4)	< 0.001	− 1.3 (3.5)	0.037
Nasion to menton	101.8 (3.6)				
Horizontal measurements					
Intercanthal distance (en-en)	32.2 (1.9)	− 1.1 (1.9)	0.002	−3.6 (1.9)	< 0.001
Eye width (ex-en)	34.0 (3.4)	2.7 (3.4)	< 0.001	1.1 (3.4)	0.071
Biocular width (ex-ex)	98.2 (3.5)	7.0 (3.5)	< 0.001	1.4 (3.5)	0.021
Nasal width (al-al)	43.2 (3.8)	8.3 (3.8)	< 0.001	− 1.0 (3.8)	0.139
Ala curvature (ac-ac)	41.3 (3.1)	8.5 (3.1)	< 0.001	1.3 (3.1)	0.019
Mouth width (ch-ch)	55.9 (3.3)	1.4 (3.3)	0.014	1.3 (3.3)	0.022
Bitemporal width (ft-ft)	115.3 (3.4)	− 0.6 (3.4)	0.273	− 1.0 (3.4)	0.077
Bizygomatic width (zy-zy)	133.8 (4,6))	− 5.3 (4.6)	< 0.001	− 4.9 (4.6)	< 0.001
Bigonial width (go-go)	106.6 (5.9)	1.0 (5.9)	0.312	2.4 (5.9)	0.019
Kenyan female face					
Vertical measurements					
Forehead height I (tr-g)	55.4 (3.3)	2.7 (3.3)	< 0.001	− 0.3 (3.3)	0.545
Forehead height II (tr-n)	67.5 (2.9)	4.5 (2.9)	< 0.001	0.4 (2.9)	0.423
Midface height (g-sn)	65.3 (5.7)	2.2 (5.7)	0.027	0.7 (5.7)	0.481
Nasal height (n-sn)	47.6 (3.1)	− 3.0 (3.1)	< 0.001	− 1.2 (3.1)	0.029
Lower face height (sn-me)	65.0 (4.8)	5.2 (4.8)	< 0.001	− 2.0 (4.8)	0.013
Upper lip height (sn-sto)	24.0 (2.5)	3.9 (2.5)	< 0.001	− 0.5 (2.5)	0.267
Lower lip height (sto-sl)	20.7 (1.1)	2.9 (1.1)	< 0.001	0.5 (1.1)	0.004
Philtrum height	13.5 (1.6)	− 0.3 (1.6)	0.229	− 0.5 (1.6)	0.052
Lateral commissure height	22.3 (2.3)				
Upper vermillion height (Is-sto)	13.4 (0.9)	4.7 (0.9)	< 0.001	0.1 (0.9)	0.332
Lower vermillion height (sto-li)	13.6 (1.0)	4.2 (1.0)	< 0.001	0.4 (1.0)	0.031
Chin height (sl-gn)	34.1 (3.2)	7.1 (3.2)	< 0.001	− 1.1 (3.2)	0.046
Lower lip to labiomental fold	10.6 (1.1)	−0.2 (1.1)	0.401	− 0.2 (1.1)	0.401
Labiomental fold	7.9 (2.8)				
Lower lip to menton	51.6 (3.4)	8.2 (3.4)	< 0.001	− 0.5 (3.4)	0.399
Nasion to menton	117.1 (5.4)				
Horizontal measurements					
Intercanthal distance (en-en)	32.1 (1.4)	0.3 (1.4)	0.136	− 2.3 (1.4)	< .001
Eye width (ex-en)	33.7 (1.5)	3.0 (1.5)	< 0.001	1.5 (1.5)	< 0.001
Biocular width (ex-ex)	94.4 (4.9)	6.6 (4.9)	< 0.001	1.5 (4.9)	0.077
Nasal width (al-al)	40.7 (3.7)	9.3 (3.7)	< 0.001	0.6 (3.7)	0.313
Ala curvature (R – L)	33.5 (2.2)	3.0 (2.2)	< 0.001	1.3 (2.2)	0.019
Mouth width (ch-ch)	52.0 (4.0)	1.8 (4.0)	0.008	− 1.6 (4.0)	0.022
Bitemporal width (ft-ft)	111.2 (2.5)	− 0.3 (2.5)	0.430	− 0.2 (2.5)	0.582
Bizygomatic width (zy-zy)	130.1 (3.5)	0.1 (3.5)	0.827	− 0.4 (3.5)	0.523
Bigonial width (go-go)	96.8 (2.9)	2.3 (2.9)	0.001	0.1 (2.9)	0.880

The greatest difference in measurements was noted for the reduced nasal height, the increased interalar width, and nasal curvature. The only measurement that demonstrated similarity was the lower lip to the labiomental fold, having a mean difference of 0.3 mm.

When compared with the African American population, only two vertical and two horizontal measurements showed a statistically significant difference. The following values are expressed as mean difference with standard deviation. The Kenyan male face had a shorter midface and lower face height, with a mean difference of − 4.0 mm (6.51) and − 2.6 mm (3.55), respectively, and philtrum height of − 0.9 mm (1.25). The two measurements that displayed significant difference were the intercanthal distance and the bizygomatic width. Clinically, no significant difference was found in the vertical measurements, but two horizontal measurements were clinically significant, bizygomatic width (− 4.9 mm) and intercanthal distance (− 3.6 mm), both being reduced in the Kenyan male face.

Across the three populations, the only measurement that showed statistically significant difference was the lower face height, which was increased compared with NAW and decreased compared with AA. The only measurement that had a clinical significant difference was observed in the Kenyan male with the bizygomatic width, which was reduced when compared with both NAW and AA males. This was not observed in the female subjects.

The Kenyan female face, when compared to the NAW, demonstrated a significant difference of 14 measurements of the 22 carried out. The forehead height ~ 2.7 mm (3.3) (*p* < 0.001), midface height ~ 2.2 mm (5.7) (*p* < 0.001), and lower face height 4.8 mm (*p* < 0.001) were increased in the Kenyan female compared to the NAW. However, compared to the AA, there was no statistical difference in the forehead ~ − 0.3 mm (3.3) (*p* = 0.545), midface ~ 0.7 mm (5.7) (*p* = 0.481), and lower face heights ~ − 2.0 mm (4.8) (*p* = 0.013). Only two measurements, intercanthal distance ~ − 2.3 mm (1.4) (*p* < 0.001) and eye width ~ 1.5 mm (1.5) (*p* < 0.001), showed a statistically significant difference.

The nasal height was reduced compared with both the NAW and AA though not statistically different when compared to the AA. Across the three populations, the only measurement of the Kenyan female face that showed a statistically significant difference was the eye width, having a mean difference of ~ 3.0 mm (1.5) NAW and ~ 1.5 mm (1.5) AA.

Overall, when both the Kenyan male face and female face were compared with the NAW, the greatest differences were found in the measurements of the reduced nasal height ~ − 3.8 mm (1.9) (*p* < 0.001) and increased nasal width ~ 8.3 mm (3.8) (*p* < 0.001), which were clinically significant. When compared with the AA, the only statistically significant difference in both males and females was the reduced intercanthal distance ~ − 3.6 mm (1.9) (*p* < 0.001) in the Kenyan population. However, this measurement was only clinically significant in the male participants.

#### Comparative data with neoclassical proportional canons

Seven neoclassical canons (Table 7) and five proportional indices (Table 8) were also investigated in the Kenyan sample. Most of the sample ratios did not comply with the neoclassical canons. When comparing the Kenyan male and Kenyan female to the neoclassical canons, the only canon which was valid for the majority of participants was the orbital canon (Canon VI). This was observed in 12 males and 10 female participants. For the vertical measurement, the forehead height exceeded the nasal height in the entire sample. Only 6% of males and 11% of females had forehead height equal to the lower facial height, with the majority demonstrating reduced lower face height compared to the forehead height. For the naso-oral canon, none of the participant’s measurements demonstrated similarity, with 6% of males and 11% of females of the participants having proportionate values (Table 9). Regarding the orbitonasal proportion, none of the participants corresponded with it, with 100% of the participants exhibiting a greater nasal width compared to the intercanthal distance. The nasofacial proportional canon demonstrated that all the participants had a nasal width greater than the quarter of the facial width (Table 7). All of the proportional indices pertaining to the Kenyan African males and females in our investigation differed significantly from the North American white population, with the greatest mean difference observed in the total upper lip height, intercanthal, and nasal width proportion (Table 8).

**Table 7:** Application of neoclassical canons to Kenyan male and female face

Canon II	KM %	KF%
tr-n = n-sn > 1	100	100
tr-n = n-sn < −1	0	0
tr-n = n-sn > = − 1 < =1	0	0
Canon II		
tr-n = sn-me> 1	8	30
tr-n = sn-me<−1	86	59
tr-n = sn-me> = −1 < =1	6	11
Canon III		
tr-g = g-sn > 1	31	3
tr-g = g-sn < −1	64	95
tr-g = g-sn > = − 1 < =1	6	3
Canon V		
en-en = al-al > 1	0	0
en-en = al-al < −1	100	100
en-en = al-al > = − 1 < =1	0	0
Canon VI		
en-en = ex-en > 1	17	8
en-en = ex-en < −1	50	65
en-en = ex-en > = − 1 < =1	33	27
Canon VII		
ch-ch = 1.5(al-al) > 1	6	3
ch-ch = 1.5(al-al) < −1	89	86
ch-ch = 1.5(al-al) > = − 1 < =1	6	11
Canon VIII		
al-al = 0.25(zy-zy) > 1	100	100
al-al = 0.25(zy-zy) < −1	0	0
al-al = 0.25(zy-zy) > = − 1 < =1	0	0

**Table 8:** Proportional indices comparison of Kenyan African males and females to North American Whites

	KAM	Diff NAW	KAF	Diff NAW	*P* value
Vermillion total upper lip height	55.9 (5.5)	12.8 (5.5)	56.3 (5.7)	27.5 (39.6)	< *0*.*001*
Vermillion cutaneous upper lip height	99.4 (11.1)	11.6 (11.1)	99.2 (4.8)	11.8 (4.8)	< *0*.*001*
Nose— mouth width	77.3 (7.0)	12.0 (7.0)	78.7 (9.1)	15.4 (9.1)	< *0*.*001*
Intercanthal nasal width	75.2 (7.0)	−19.9 (7.7)	79.5 (7.4)	−21.4 (7.4)	< *0*.*001*
Lower face height	50.1 (2.4)	6.4 (2.4)	40.7 (2.4)	−3.1 (2.4)	< *0*.*001*

*KAM* Kenyan African males, *KAF* Kenyan African females, *NAW* North American Whites

**Table 9:** Descriptive statistics of measurements of the Kenyan African male and Kenyan African female

	Male		Female	
	Mean	Standard deviation ±	Mean	Standard deviation ±
Vertical measurements				
Forehead height I (tr-g)	61.4	3.2	55.4	3.3
Forehead height II (tr-n)	72.2	2.3	67.5	2.9
Midface height (g-sn)	64.8	6.5	65.3	5.7
Nasal height (n-sn)	51.0	1.9	47.6	3.1
Lower face height (sn-me)	76.4	3.6	69.5	4.8
Upper lip height (sn-sto)	25.5	1.3	24.0	2.5
Lower lip height (sto-sl)	22.5	1.9	20.7	1.1
Philtrum height (sn-ls)	15.5	1.3	13.5	1.6
Lateral commissure height	23.5	2.6	22.3	2.3
Upper vermillion height (Is-sto)	13.7	1.3	13.4	0.9
Lower vermillion height (sto-li)	13.8	0.9	13.6	1.0
Chin height (fml-me)	36.5	3.1	34.1	3.2
Lower lip to labiomental fold	12.1	1.9	10.6	1.1
Labiomental fold	8.2	1.9	7.9	2.8
Lower lip to menton	56.2	3.5	51.6	3.4
Nasion to menton	101.8	3.6	117.1	5.4
Horizontal measurements				
Intercanthal distance (en-en)	32.2	1.9	32.1	1.4
Eye width (ex-en)	34.0	3.4	33.7	1.5
Biocular width (ex-ex)	98.2	3.5	94.4	4.9
Nasal width (al-al)	43.2	3.8	40.7	3.7
Ala curvature (ac-ac)	41.3	3.1	33.5	2.2
Mouth width (ch-ch)	55.9	3.3	52.0	4.0
Bitemporal width (ft-ft)	115.3	3.4	111.2	2.5
Bizygomatic width(zy-zy)	133.8	4.6	130.1	3.5
Bigonial width (go-go)	106.6	5.9	96.8	2.9

## Discussion

Ethnic variability should always be considered during diagnosis and treatment planning of orthognathic or craniofacial reconstructive treatment. Treating subjects from different ethnic groups using normative anthropometric data from another group, or neoclassical canons, for comparison may be misleading and inaccurate [1, 14, 15].

Clinicians may be faced with the predicament of how to make a distinction between normal and abnormal in a patient’s face, due to the presence of a large number of variables [22]. These consist of but are not limited to age, gender, ethnicity, and cultural perceptual variability. In most cases, it is deemed as imperative to treat patients to what constitutes as typical or average for their population, specific for age, gender, and ethnic background. This forms the rationale for establishing normative anthropometric data [1].

Farkas carried out the prevalent comparative studies on intercontinental populations and verified contrasts in the average faces when compared to neoclassical canons [5, 13, 18]. Various other researchers have carried out similar studies on Indian [6], Iranian [23], Turkish [9], Chinese [10], and African American populations [3, 5].

There are numerous methods utilized to obtain anthropometric information, including indirect methods such as photogrammetry and more recently 3D scanning photogrammetry. However, even with progress, these methodologies may still be considered potentially inferior to direct anthropometric measurements [1, 4]. The main drawbacks to 3D imaging are the expense and complexity of the equipment, the time-consuming processes required to produce images, and the risk of error if subjects are not stationary through the scanning process [24, 25]. Errors in software and its utilization may also be relevant factors.

Photogrammetric studies have the advantage of being simpler to conduct as they avoid direct measurements of facial soft tissue and hence may reduce the likelihood of error due to soft tissues displacement [16]. However, photogrammetric measurements are known to be less accurate than direct measured anthropometric analysis [17]. One indirect photogrammetric measurement study carried out comparing Kenyans with Chinese has been described in the literature and demonstrated many differences in average angular measurements of the facial profiles of black Kenyans, Chinese, and white standards [12]. Nevertheless, it is also recognized that direct facial soft tissue anthropometric measurement can be difficult and time-consuming due to the “give” or minor sinking of soft tissue when the measuring instruments are positioned on the facial landmarks [16].

A systematic review utilizing pooled data from studies of various ethnic groups concluded that the height of the forehead, eyes, nose, and mouth exhibited the greatest interethnic variability [26]. In the current investigation, the anthropometric measurements of both the Kenyan African males and females revealed that the facial characteristics of the population studied varied notably from the North American white subjects. The study further confirmed some similarities to the African American population. When comparing the Kenyan African male to the North American whites, 8 of the 10 measurements were clinically significantly different based on the two-sample *t* test, and 13 of the 22 measurements were clinically significantly different compared with the one-sample *t* test. Repeated measures can introduce the likelihood of a type 1 error.

The large number of significantly different proportions with a *p* value of < 0.001 demonstrated that this population differed from the NAW. The greatest difference was observed in the reduced nasal height, the increased nasal width, and increased nasal curvature, with the only parameter that was similar between all three populations being the mouth width (ch-ch). A similar trend regarding the labio-oral region being identical was observed in 12 of 13 Caucasian groups, 4 of 5 Asian groups, and all Middle Eastern and African ethnic groups in an international study [16].

When compared to the African American population, the data in this investigation demonstrated no clinically significant differences except for intercanthal distance being reduced in the Kenyan males. However, this finding was significant and distinctive as other studies on African males from Tonga, Angola, Zulu, and African Americans have all been observed intercanthal distance to be identical to NAW [11, 16].

Similar results were obtained with the female data with forehead height being greater than the North American whites, though similar to the African Americans. This has also been observed in the Sudanese female face with greater forehead height compared to NAW and AA [16]. The nasal height was shorter for Kenyan African females, though slightly increased compared with African Americans, but this was not significantly different (*p* = 0.693). The nasal width and curvature were greater compared to the North American whites. Between the groups, mouth width was similar and when compared to the African American female, the Kenyan females had greater eye width and intercanthal distance (*p* < 0.001).

The nasolabial and labiomental angular measurements of the Kenyan African male had a clinically significant difference of 10° when compared with the North American white and the African American subjects. In the Kenyan females, only the nasolabial angle had a clinically significant difference when compared with both populations, with only the labiomental angle exhibiting a clinical significant difference to the AA female.

When comparing Kenyan faces to Chinese faces, in a photogrammetric study, the only comparable angle was the facial convexity, which was also similar to NAW. The nasal dorsum and lower face height were also comparable in both populations, with all other angular measurements showing large ethnic differences [12].

Despite there only being a difference of 1–2 mm between some of the measurements, the overall data does propose that the Kenyan population does have a considerable difference in comparison to North American whites and have comparative facial features to the African American populations except for the reduced intercanthal distance observed in the male participants only.

In this investigation, both the Kenyan males and females had reduced intercanthal distance (en-en) compared with the eye fissure length. This was in contrast to the observations in Farkas’ international study, where the intercanthal distance was wider than the eye fissure length in the African Americans [20]. The most significant variation was regarding the orbitonasal proportional canon, as none of the participants corresponded with it. In this investigation, the nasofacial proportional canon demonstrated that all the participants had a nasal width greater than a quarter of the facial width. All of the participants exhibited a greater nasal width compared to the intercanthal distance. The Kenyan naso-orbital proportion was similar to the African Americans (94%) population.

The period coinciding with the European Enlightenment gave rise to the neoclassical proportional canons, which were reworkings based on classical canons [1]. These measurements were predominantly important for artists [27, 28]. The era of the 17th and 18th centuries were immensely influenced by the neoclassical canons, with their influence diminishing by the nineteenth century. Currently, they remain as a classical foundation around which some of modern-day facial analysis is based [1]. However, the results of modern anthropometric studies, and facial attractiveness studies, may update such canons for the modern day [1, 11].

In the Kenyan sample, the neoclassical canons of facial proportion were not applicable. This has been observed in similar investigations on African American males and African American females [17]. The vertical facial trisection canon for upper, middle, and lower facial heights being equal thirds was not observed. The middle third of the face was identified as being the smallest of the three proportions. The most frequently valid canon tested was the orbital canon, being valid in 33% of the males and 27% of the females, which was comparable to previous studies [8, 13].

The anthropometric data from this investigation, in terms of linear measurements (Table 9), angular measurements (Tables 4 and 5), and proportional values (Table 8) described, provides a potentially valuable data set, and could serve as a database for facial analysis in the Kenyan African population.

## Conclusion

This is the first anthropometric study on Kenyan males and females, testing the validity of the neoclassical canons and providing a database for the average horizontal and vertical measurements and proportions of the population.

Young adult Kenyan males and females were chosen for this investigation because they form the main ethnic group in Kenya. The participants were ethnic Kenyans studying at the University of Nairobi and within the limitations of this study the normative data provided may be used to represent the Kenyan normative values.

In general, it was observed that both the Kenyan males and females had a trend for an increased forehead height (~ 5 mm) compared to the reduced middle third of the face and reduced nasal height (~ 4 mm), and taller lower face (~ 4–5 mm). The most distinguishing feature was the increased nasal width (~ 8 mm) and wider eye fissure length compared to the intercanthal distance. Upper lip height was also significantly greater in the Kenyan population (~ 3–4 mm). Despite the previously reported differences of other African ethnic groups, such as Sudanese females, the Kenyan population sampled in this investigation had comparable facial features to the African American populations, except for the reduced intercanthal distance observed in the male participants only.

None of the neoclassical canons were valid for this group of young Kenyan adults. This study does verify that anthropometric measurements of Caucasian populations are invalid when applied to the Kenyan population, and variations do exist in comparison with African American normative data. It is recommended that accurate and applicable data is used in diagnosis and treatment planning for each ethnic group.
